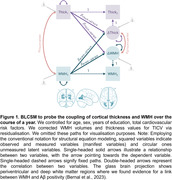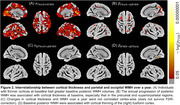# Cortical neurodegeneration influences annual posterior white matter hyperintensity progression

**DOI:** 10.1002/alz.086414

**Published:** 2025-01-09

**Authors:** Jose Bernal Moyano, Inga Menze, Renat Yakupov, Oliver Peters, Josef Priller, Anja Schneider, Klaus Fliessbach, Jens Wiltfang, Frank Jessen, Katharina Buerger, Robert Perneczky, Stefan Teipel, Christoph Laske, Annika Spottke, Michael T. Heneka, Stefanie Schreiber, Emrah Düzel, Gabriel Ziegler

**Affiliations:** ^1^ Institute of Cognitive Neurology and Dementia Research (IKND), Otto‐von‐Guericke University, Magdeburg Germany; ^2^ German Center for Neurodegenerative Diseases (DZNE), Magdeburg Germany; ^3^ Charité – Universitätsmedizin Berlin, corporate member of Freie Universität Berlin and Humboldt‐Universität zu Berlin – Institute of Psychiatry and Psychotherapy, Berlin Germany; ^4^ German Center for Neurodegenerative Diseases (DZNE), Berlin Germany; ^5^ Department of Psychiatry and Psychotherapy, Technical University of Munich, Munich Germany; ^6^ University of Edinburgh and UK DRI, Edinburgh UK; ^7^ Department of Psychiatry and Psychotherapy, Charité, Charitéplatz 1, Berlin Germany; ^8^ German Center for Neurodegenerative Diseases (DZNE), Bonn, Venusberg‐Campus 1, 53127 Bonn, Germany, Bonn Germany; ^9^ University of Bonn Medical Center, Dept. of Neurodegenerative Disease and Geriatric Psychiatry/Psychiatry, Venusberg‐Campus 1, 53127 Bonn, Germany, Bonn Germany; ^10^ German Center for Neurodegenerative Diseases (DZNE), Goettingen Germany; ^11^ Department of Psychiatry and Psychotherapy, University Medical Center, University of Goettingen, Goettingen Germany; ^12^ Neurosciences and Signaling Group, Institute of Biomedicine (iBiMED), Department of Medical Sciences, University of Aveiro, Aveiro Portugal; ^13^ German Center for Neurodegenerative Diseases (DZNE), Bonn Germany; ^14^ Excellence Cluster on Cellular Stress Responses in Aging‐Associated Diseases (CECAD), University of Cologne, Cologne Germany; ^15^ Department of Psychiatry, University of Cologne, Medical Faculty, Cologne Germany; ^16^ Institute for Stroke and Dementia Research (ISD), University Hospital, LMU Munich, Munich Germany; ^17^ German Center for Neurodegenerative Diseases (DZNE), Munich Germany; ^18^ LMU University Hospital, Munich Germany; ^19^ German Center for Neurodegenerative Diseases (DZNE, Munich), Feodor‐Lynen‐Strasse 17, 81377 Munich, Germany, Munich Germany; ^20^ Munich Cluster for Systems Neurology (SyNergy), Munich Germany; ^21^ Department of Psychiatry and Psychotherapy, Klinikum der Ludwig‐Maximilians Universität München, Munich Germany; ^22^ German Center for Neurodegenerative Diseases (DZNE), Rostock Germany; ^23^ Department of Psychosomatic Medicine, University of Rostock, Rostock Germany; ^24^ German Center for Neurodegenerative Diseases (DZNE), Tuebingen Germany; ^25^ Section for Dementia Research, Hertie Institute for Clinical Brain Research and Department of Psychiatry and Psychotherapy, University of Tuebingen, Tuebingen Germany; ^26^ Department of Neurology, University of Bonn, Bonn Germany; ^27^ Luxembourg Centre for Systems Biomedicine (LCSB), University of Luxembourg, Luxembourg Luxembourg; ^28^ Department of Neurology, Otto‐von‐Guericke University, Magdeburg Germany

## Abstract

**Background:**

For over three decades, the concomitance of cortical neurodegeneration and white matter hyperintensities (WMH) has sparked discussion about their coupled temporal dynamics (Garnier‐Crussard et al. 2023). Longitudinal evidence supporting this hypothesis remains nonetheless scarce (Ter Telgte et al. 2018).

We integrated surface‐based morphometry and bivariate latent change score modelling (BLCSM) to examine interrelationships between individual WMH and cortical thickness changes over a one‐year period in cognitively unimpaired participants.

**Method:**

We analysed baseline and 12‐month follow‐up data from cognitively unimpaired DELCODE participants (n=393; median age 70.31 [IQR 66.06‐74.87] years; 52% females). We used T2w FLAIR and T1w MPRAGE data to segment WMH (LST; Schmidt and Wink 2019) and estimate cortical thicknesses (CAT12; Gaser et al., n.d.).

Using BLCSM in a vertex‐wise fashion, we tested whether baseline WMH volumes predicted cortical thinning rates and whether baseline cortical thickness predicted WMH volumes increases (Figure 1). Due to the posterior dominance of WMH in AD (Bernal et al. 2023), we focussed on parietal and occipital WMH. All models included age, sex, years of education, and total cardiovascular risk factors as covariates on baseline and change scores. We log‐10 transformed WMH volumes and corrected WMH volumes and thicknesses measurements for TICV via residualisation. We finally tested for moderation effects of CSF‐derived Aβ42/40 and pTau181 on cross‐domain coupling.

**Result:**

BLCSM generally provided good data fits (RMSEA≤0.05, CFI≥0.095, SRMR≤0.05) across all analyses.

The mean thickness across precuneal and superiorparietal cortices at baseline predicted the progression of WMH better than any other cortical region (Figure 2B; β_Thick→ΔWMH_=‐0.051, SE=0.011, Z=‐4.535, p‐value<0.001). This was especially evident with lower CSF‐derived Aβ42/40 (β_Thick*Aβ42/40→ΔWMH_=0.176, SE=0.053, Z=3.309, p‐value=0.001).

WMH volume at baseline explained, in part, the level of thinning of parts of the fusiform cortex (Figure 2D; β_WMH→ΔThick_=‐0.139, SE=0.028, Z=‐5.030, p‐value<0.001). This interrelationship was moderated by CSF‐derived pTau181 (β_WMH*pTau181→ΔThick_=‐0.144, SE=0.053, Z=‐2.711, p‐value=0.007).

**Conclusion:**

Cortical thinning and WMH progression may be mutually reinforcing processes that become reciprocally coupled prior to any detectable cognitive deficits. Hallmark AD proteins appear to moderate their interrelationships.